# Mesenchymal Stem Cell-Specific and Preosteoblast-Specific Ablation of TSC1 in Mice Lead to Severe and Slight Spinal Dysplasia, Respectively

**DOI:** 10.1155/2020/4572687

**Published:** 2020-03-26

**Authors:** Cheng Yang, Jianwen Liao, Pinglin Lai, Hai Huang, Shicai Fan, Yuhui Chen, Xiaochun Bai

**Affiliations:** ^1^Academy of Orthopedics, Guangdong Province, Department of Orthopedic Surgery, The Third Affiliated Hospital of Southern Medical University, Guangzhou 510630, China; ^2^Department of Cell Biology, School of Basic Medical Science, Southern Medical University, Guangzhou 510515, China

## Abstract

**Background:**

TSC1-related signaling plays a pivotal role in intramembranous and endochondral ossification processes during skeletogenesis. This study was aimed at determining the significance of the TSC1 gene at different stages of spinal development. *Materials and Methods*. TSC1-floxed mice (TSC1^flox/flox^) were crossed with Prrx1-Cre or BGLAP-Cre transgenic mice or mesenchymal stem cell- and osteoblast-specific TSC1-deficient mice, respectively. Somatic and vertebral differences between WT and Prrx1-TSC1 null mice were examined at 4 weeks after birth.

**Results:**

No apparent body size abnormalities were apparent in newborn and 4-week- to 2-month-old mice with BGLAP-Cre driver-depleted TSC1. Vertebral and intervertebral discs displayed strong dysplasia in Prrx1-TSC1 null mice. In contrast, vertebrae were only slightly affected, and intervertebral discs from skeletal preparations displayed no apparent changes in BGLAP-TSC1 null mice.

**Conclusion:**

Our data suggest that the TSC1 gene is crucial for endochondral ossification during postnatal spine development but plays discriminative roles at different stages. Mesenchymal stem cell-specific ablation of TSC1 led to severe spinal dysplasia at early stages of endochondral ossification while osteoblast-specific deletion of TSC1 affected vertebrae slightly and had no detectable effects on intervertebral discs.

## 1. Introduction

Endochondral and intramembranous ossifications are two types of skeletal ossification processes [1]. Endochondral ossification is the dominant pathway of limb and vertebral growth after embryonic development [2, 3]. During this process, mesenchymal stem cells (MSC) in the cartilaginous template differentiate into chondrocytes, following which the chondrification centers are eventually replaced with bone-containing osteoblasts and osteoclasts [4]. Classically, disruption of spinal endochondral ossification can result in spinal dysplasia and consequent development of congenital spinal deformities [5–7]. However, the mechanisms underlying spinal endochondral ossification are yet to be clarified.

Mammalian target of rapamycin complex 1 (mTORC1) uniquely contains raptor and controls protein synthesis through phosphorylation of eukaryotic initiation factor 4E–binding protein-1 (4E-BP1) and S6 kinase 1 (S6K1) [8–10]. In response to energy switch or stress, mTORC1 is activated by two families of ras-related small guanosine triphosphatases (GTPase), Rheb and Rags [11]. GTP-bound (active) Rheb is suppressed by tuberous sclerosis complex 1/2 (TSC1/2), a functional complex displaying GTPase-activating protein (GAP) activity, and the deletion of TSC1 triggers constitutive mTORC1 activation [8–11]. In recent studies, the mTORC1 pathway has been implicated as a key modulator of BMSC fate and osteoblastic differentiation [8–11]. Interestingly, however, mTORC1 inhibition with rapamycin or gene ablation is reported to exert either stimulatory or inhibitory effects on osteoblastic differentiation and endochondral ossification [12–17]. These conflicting results may be attributable to distinct roles of mTORC1 at various stages of osteoblastic differentiation in endochondral ossification [2]. Recent experiments by our group showed that overactivation of mTORC1 in osterix-positive preosteoblasts prevents osteoblastic differentiation [18]. Similarly, overactivation of mTORC1 signaling in collagen-II positive chondrocytes resulted in retardation of endochondral ossification and congenital spinal deformities [19]. However, owing to the complexity of mechanisms underlying osteoblastic differentiation in endochondral ossification, the stage-specific roles of mTORC1 on bone development remain unclear.

In the present study, Cre/LoxP technology was applied to delete tuberous sclerosis 1 (TSC1, a major upstream inhibitory regulator of mTORC1) in Prrx-1-positive mesenchymal stem cells and osteocalcin (BGLAP)-positive preosteoblasts in mice and postnatal spinal development examined, with a view to determining the functions of TSC1 in MSCs and preosteoblasts involved in spinal endochondral ossification.

## 2. Materials and Methods

### 2.1. Animals

To specifically delete TSC1 in mesenchymal stem cells and osteoblasts, we crossed TSC1-floxed mice (TSC1^flox/flox^ Jackson #005680) with Prrx1-Cre (Jackson #005584) or BGLAP-Cre (Jackson #019509) mice to generate conditional TSC1 knockout mice according to classic mice cross^19^. TSC1^+/-^, Prrx1-Cre or TSC1^+/-^, and BGLAP-Cre mice were mated, and TSC1^flox/flox^, Prrx1-Cre and TSC1^flox/flox^, and BGLAP-Cre mice were selected as the experimental groups (KO). TSC1^+/+^, Prrx1-Cre or TSC1^+/+^, and BGLAP-Cre littermates served as controls (WT). Newborn mice were analyzed via PCR genotyping of tail genomic DNA. All animal experiments were approved by the Ethical Committee of Southern Medical University (Guangzhou, China).

### 2.2. Skeletal Preparations

Whole-mount skeletal preparations were prepared by removing the skin and internal organs of mice before immersion in 95% ethanol overnight. Specimens were stained with 0.015% Alcian Blue 8GX (Sigma-Aldrich, St Louis, MO, USA) in 80% ethanol/20% acetic acid and 0.005% Alizarin Red S (Sigma-Aldrich) in 1% KOH after digestion with 2% KOH overnight. Next, specimens were cleared with 1% KOH/20% glycerol solution and stored in a 1 : 1 mixture of glycerol and 95% ethanol.

### 2.3. Micro-CT Analysis

Tomography of fixed spine specimens was performed using a microtomographic (microcomputed tomography, micro-CT) imaging system (ZKKS-MCT-Sharp-III scanner, Caskaishen, China). A small field was selected for scanning and corrected for CT values, with 70 kV scanning voltage, 30 W power, 429 *μ*A current, and 5 *μ*m scan thickness. 3D-MED 3.0 was applied for three-dimensional spine reconstruction and image capture.

### 2.4. Histological Analysis

Formalin-fixed samples were decalcified in 20% EDTA (pH 7.2) for three weeks, dehydrated in a graded series of ethanol, cleared, and embedded in paraffin. Sagittal sections (5 *μ*m) were cut and stained with hematoxylin and eosin (H&E) and Alcian blue (Sigma-Aldrich) [19].

### 2.5. Statistical Analysis

Data were expressed as means ± SD. Two-tailed Students' *t* test and one-way ANOVA test were performed for analysis of statistical significance. Data from representative experiments are presented in Results and corresponding figures. Statistical significance was achieved at *p* < 0.05.

## 3. Results

### 3.1. Appearance and Body Length Are Altered in Prrx-1-TSC1 Null but Not BGLAP-TSC1 Null Mice during Development

**Figure 1:**
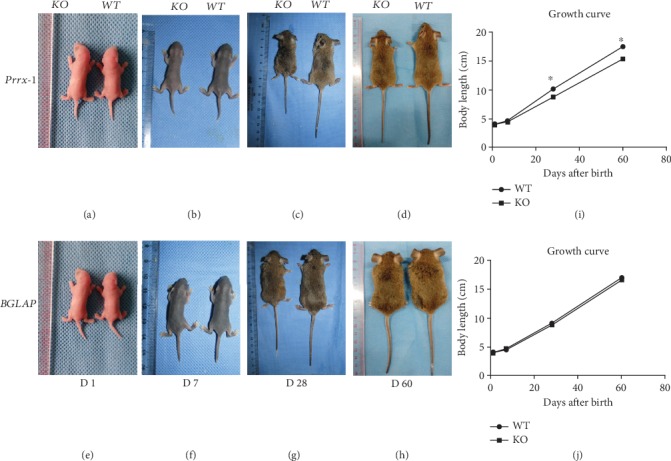
Skeletal phenotypes of WT, Prrx1-TSC1 Null, and BGLAP-TSC1 null mice. (a-d) Appearance of developing WT and Prrx1-TSC1 null mice over different periods. (e-h) Appearance of developing WT and BGLAP-TSC1 null mice over different periods. (i, j) Body lengths of mice at different times (*N* = 6, “^∗^” represents *p* < 0.05).

The body lengths of TSC1^+/+^-Prrx1-Cre (WT) and Prrx1-TSC1 null mice were measured on days 1, 7, 28, and 60 after birth. As shown in Figure 1, no appearance alterations in BGLAP-TSC1 and Prrx1-TSC1 null mice relative to their WT counterparts were observed on days 1 and 7 (Figures 1(a), 1(b), and 1(i)). However, the body lengths of Prrx1-TSC1 null mice appeared significantly decreased, compared with WT mice, on days 28 and 60 after birth (Figures 1(c), 1(d), and 1(i)). In contrast, no skeletal abnormalities and body length changes were apparent in BGLAP-TSC1 null mice from the newborn stage to day 60, compared with WT mice (Figures 1(e)–1(h), and 1(j)).

### 3.2. Bony Spinal Dysplasia Is Severe in Prrx1-TSC1 Null Mice and Absent in BGLAP-TSC1 Mice

**Figure 2:**
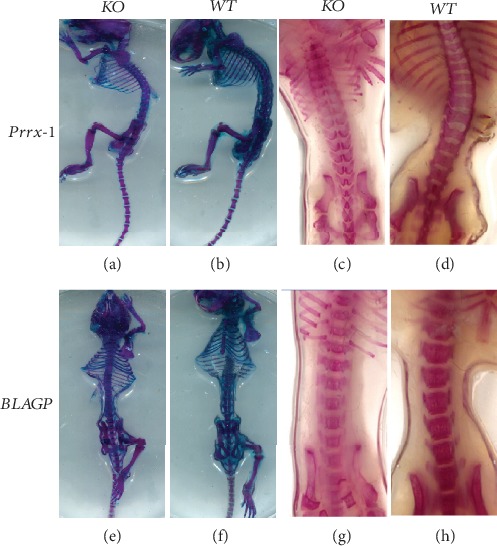
Skeletal staining of WT, Prrx1-TSC1 null, and BGLAP-TSC1 null mice eight weeks after birth. (a, b) Alcian blue staining of the skeletons of WT and Prrx1-TSC1 null mice. (c, d) Alizarin staining of the skeletons of WT and Prrx1-TSC1 null mice. (e, f) Alcian blue staining of the skeletons of WT and BGLAP-TSC1 null mice. (g, h) Alizarin staining of the skeletons of WT and BGLAP-TSC1 null mice.

To assess spinal development, Alizarin and Alcian blue staining of the skeleton was performed in Prrx1-TSC1 null and BGLAP-TSC1 null mice at 8 weeks after birth. While no significant changes in intervertebral discs and number of vertebrae were evident in Prrx1-TSC1 null mice, spine length was remarkably decreased, compared with that of WT mice (Figures 2(a) and 2(b)). Moreover, individual vertebrae were smaller and shorter (Figures 2(c) and 2(d)). The spines of all Prrx1-TSC1-null mice were unfused, and vertebrae had formed a wedge structure, which was low in the middle but high on both sides (Figures 2(c) and 2(d)). Spinal development was not complete in both coronal and sagittal planes. The cortex of vertebrae was evidently immature in Prrx1-TSC1 null mice (Figures 2(a)–2(d)). In contrast, compared with WT mice, the width and height of single vertebrae and intervertebral discs did not appear altered in BGLAP TSC1 null mice. Meanwhile, both cortical and cancellous bone appeared mature in BGLAP-TSC1 null mice (Figures 2(e)–2(h)). Our collective results suggest that the TSC1 gene in MSCs plays a vital role in spinal development while exerting no significant effect on mature osteoblasts.

### 3.3. Bone Mass and Vertebral Height Are Altered Significantly in Prrx1-TSC1 Null and Slightly in BGLAP-TSC1 Null Mice

**Figure 3:**
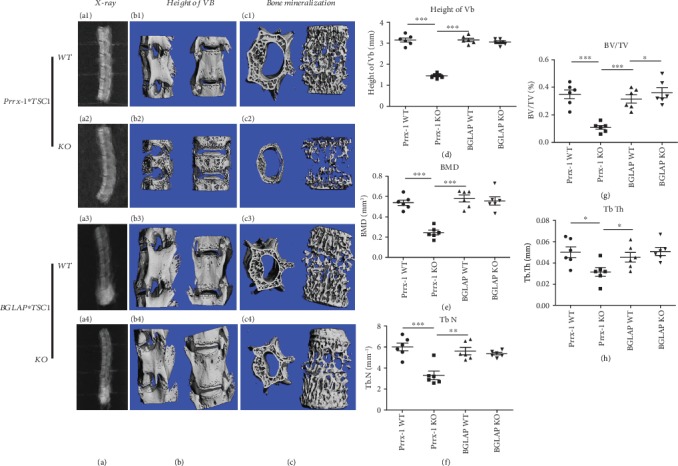
Micro-CT and bone density of WT, Prrx1-TSC1 null, and BGLAP-TSC1 null mice. (A1-A2, B1-B2, C1-C2) Micro-CT three-dimensional reconstruction and bone density of spine in WT and Prrx1-TSC1 null mice. (A3-A4, B3-B4, C3-C4) Micro-CT three-dimensional reconstruction and bone density of spine in WT and BGLAP-TSC1 null mice. (D) Statistical analysis of VB height in WT, Prrx1-TSC1 null, and BGLAP-TSC1 null mice. (E) Statistical analysis of BMD in WT, BGLAP-TSC1, and Prrx1-TSC1 null mice. (F) Statistical analysis of TB-N in WT, BGLAP-TSC1, and Prrx1-TSC1 null mice. (G) Statistical analysis of BV/TV in WT and Prrx1-TSC1 null mice. (H) Statistical analysis of Tb-Th in WT and Prrx1-TSC1 null mice. (*n* = 6, “^∗^” represents *p* < 0.05; “^∗∗^” represents *p* < 0.01; “^∗∗∗^” represents *p* < 0.001).

To assess height and bone mass of vertebrae, micro-CT tests were performed on the lumber vertebrae (L1-L6) of WT, Prrx1-TSC1 null, and BGLAP-TSC1 null mice on postnatal week 8. In Prrx1-TSC1 null mice, a significant decline in vertebral height was evident relative to WT mice in addition to decreased BMD, BV/TV, Tb. n, and Tb. Th (Figures 3(A1), 3(A2), 3(B1), 3(B2), 3(C3), 3(C4), and 3(D)–3(H)).

Despite enhancement of BV/TV of vertebrae was presented in BGLAP-TSC1 null mice, compared to their WT counterparts (Figures 3(C3), 3(C4), and 3(G)). BMD, Tb. Th, and Tb. N of vertebrae were comparable between BGLAP-TSC1 null and WT mice (Figures 3(C3), 3(C4), 3(E), 3(F), and 3(H)). Moreover, no significant alterations in vertebral height were observed in BGLAP-TSC1 null mice relative to the WT group (Figures 3(A3), 3(A4), 3(B3), 3(B4), and 3(D)). These findings support a regulatory role of the TSC1 gene in mesenchymal stem cells in spinal development but not that in preosteoblasts.

### 3.4. TSC Depletion Induces Severe Histological Spinal Dysplasia in Prrx1-TSC1 Null Mice but Minimal Effects in BGLAP-TSC1 Null Mice

**Figure 4:**
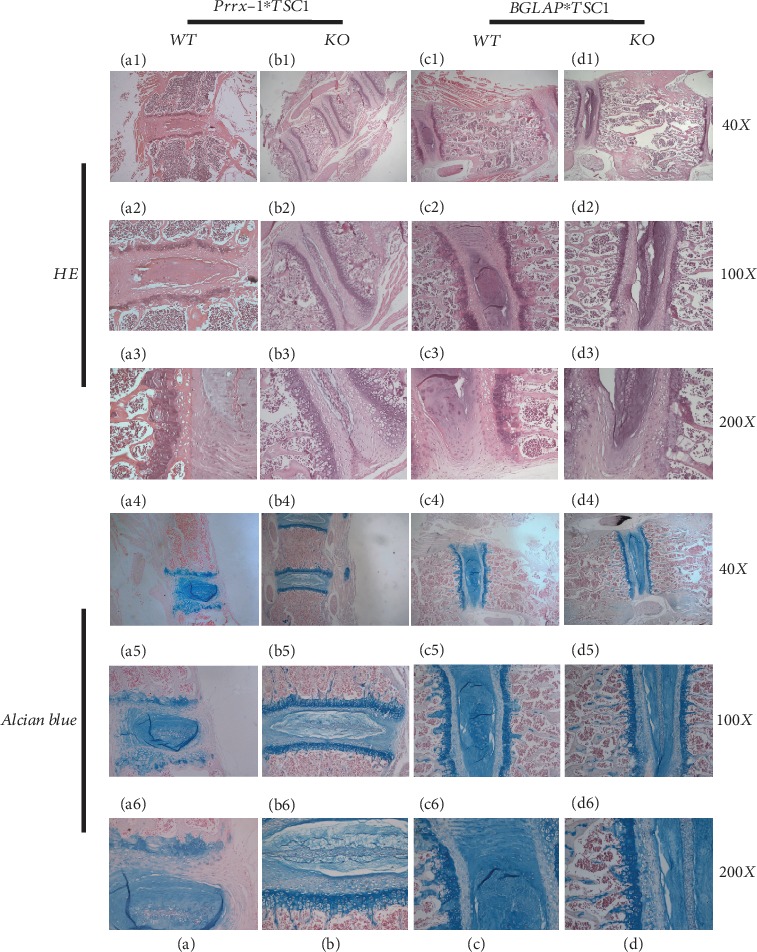
HE and Alcian blue staining of intervertebrae in Prrx1-TSC1 null, BGLAP-TSC1 null, and WT mice. (A1-A3, B1-B3) HE staining of intervertebrae in WT and Prrx1-TSC1 null mice. (A4-A6, B4-B6) Alcian blue staining of intervertebrae in Prrx1-TSC1 null and WT mice. (C1-C3, D1-D3) HE staining of intervertebrae in WT and BGLAP-TSC1 null mice. (C4-C6, D4-D6) Alcian blue staining of intervertebrae in BGLAP-TSC1 null and WT mice.

HE and Alcian blue staining were performed on Prrx1-TSC1 null, BGLAP-TSC1 null, and WT mice at 8 weeks after birth. In Prrx1-TSC1 null mice, the thickness of vertebral growth plate, cartilage endplate, and trabecular bone were remarkably decreased in addition to vertebral height (Figures 4(A1)–4(A3) and 4(B1)–4(B3)). In Alcian blue staining experiments, the majority of growth plate chondrocytes were maintained in the hypertrophy stage and immature nucleus observed in Prrx1-TSC1 null mice (Figures 4(A4)–4(A6) and 4(B4)–4(B6)). In view of the obvious calcification of growth plates and structured nucleus in WT mice, Prrx1-TSC1 null mice clearly present with delayed development and dysplasia of spine.

In contrast, the vertebral heights of BGLAP-TSC1 null mice were similar to that of WT mice. In this group, the structures of growth plates and intervertebral discs were well arranged, and the cartilage endplate, fiber ring, and nucleus pulposus showed no differences from those of WT mice (Figures 4(C1)–4(C3) and 4(D1)–4(D3)). However, Alcian blue staining revealed numerous blue trabecular bones in the middle of vertebrae, signifying immature bone tissue and retardation of mineralization (Figures 4(C4)–4(C6) and 4(D4)–4(D6)).

Taken together, our results suggest that knockout of the TSC1 gene in mesenchymal stem cells leads to severe spinal dysplasia while ablation in preosteoblasts exerts relatively minor effects.

## 4. Discussion

As a vital regulator of cell metabolism and proliferation, mTORC1 is ubiquitously expressed in all mammalian cell types [20]. A key role of mTORC1 in bone formation was originally identified in almost all skeleton-related cell lineages. However, conflicting findings regarding the precise mechanisms and functional effects of mTORC1 have been reported. Recent studies suggest that rapamycin inhibits the early stages of osteoblast differentiation in BMSCs and preosteoblasts [21]. Other experiments have demonstrated overactivation of mTORc1 in both BMSCs and preosteoblast-maintained adult bone mass *in vivo* and *in vitro* [15, 22].

Here, we hypothesized that mTORC1 is highly expressed in BMSc during the first phase of osteoblastogenesis and weakly downregulated in the last phase of mature osteoblast formation. Upon deletion of TSC1, mTORC1 activity was elevated significantly in BMSCs but only slightly in mature osteoblasts. As hyperactive mTORC1 may impair osteoblastic differentiation [18], spinal dysplasia was more marked in Prrx1-TSC1 null than in BGLAP-TSC1 null mice. Indeed, research has shown that the earlier modulation of mTORC1expression in osteoblasts, once differentiated, is associated with more significant of spinal development [18]. Experiments in the current study focused on illustrating the potential significance of the TSC1 gene at different stages of spinal development.

Previously, we reported that specific ablation of TSC1 in chondrocytes leads to retardation of chondrocyte hypertrophic and terminal differentiation [19]. Moreover, congenital spinal deformities of flat chest and thoracolumbar kyphosis were observed in chondrocyte-specific TSC1 null mice [23]. This group of mice further developed chronic wasting and seizures associated with premature death, potentially attributable to leakage of Cre in noncartilaginous tissues [24]. Similar to chondrocyte-specific TSC1 null mice, premature death at postnatal days 60-90 was recorded in both Prrx1-TSC1 null and BGLAP-TSC1 null mice. However, the specific causes of death remain to be established.

During mammalian spine development, the chondrification center appears at the embryonic stage and is transformed into chondrified vertebrae continuously at birth [25, 26]. Next, endochondral ossification manipulates spinal development gradually after birth and contributes to spinal growth until skeletal maturation [27]. In our experiments, although both Prrx1-TSC1 null and BGLAP-TSC1 null mice presented normal spine at birth, spinal dysplasia developed at postnatal week 4. In keeping with previous studies, our data suggest that overactivation of mTORC1 in chondrocytes leads to embryonic chondral dysplasia that finally results in congenital spinal deformity while overactivation at the preosteoblastic lineage disrupts postnatal endochondral ossification, triggering spinal dysplasia [23]. These findings provide indications of the specific roles of TSC1 in spinal development, both timely and spatial.

Spinal scoliosis is the most common congenital spinal deformity in the clinic followed by kyphosis [28, 29]. In our experiments, despite shortened vertebrae and spine, Prrx1-TSC1 null and BGLAP-TSC1 null mice exhibited neither scoliosis nor kyphosis. While this may be derived from premature death, compared with the congenital spinal kyphosis of Col2-TSC1 null mice reported previously by our group, this phenomenon suggests that overactivation of mTORC1 in MSCs or preosteoblasts may not contribute to spinal deformity.

In summary, we have focused on the potential significance of TSC1 gene in osteoblastic lineage cells and spinal development in this study. Deletion of the TSC1 gene in both Prrx1-positive and BGLAP-positive cell osteoblastic lineages, which target osteoblast precursors, resulted in spinal dysplasia. Interestingly, the extent of spinal dysplasia was severe in Prrx1-TSC1 null mice but relatively slight in BGLAP-TSC1 null mice, suggesting that TSC1 gene plays different roles in diversiform osteoblast precursors for mammalian endochondral ossification. Further studies are required for comprehensive understanding of the mechanisms underlying this phenomenon.

## Data Availability

All data generated or analysed during this study are included in this published article.
